# miR-484 is associated with disease recurrence and promotes migration in prostate cancer

**DOI:** 10.1042/BSR20191028

**Published:** 2020-05-07

**Authors:** Daniel Lee, Wei Tang, Tiffany H. Dorsey, Stefan Ambs

**Affiliations:** 1 Medical Oncology Service and the Center for Cancer Research, National Cancer Institute, National Institutes of Health, Bethesda, MD, U.S.A.; 2 Laboratory of Human Carcinogenesis, Center for Cancer Research, National Cancer Institute, National Institutes of Health, Bethesda, MD, U.S.A.

**Keywords:** microRNA, oncogenesis, prostate cancer

## Abstract

BACKGROUND: microRNAs (miRs) regulate the expression of protein-coding genes and play key roles in various biological processes, including development and immunity. However, dysregulation of miR expression is also involved in disease biology, including cancer.

METHODS: We utilized The Cancer Genome Atlas (TCGA) and other publicly available databases for miRs and mRNA expression in prostate cancer, selected miR-484 and investigated its role in prostate cancer biology and disease progression using *in vitro* studies.

RESULTS: Our data mining efforts revealed that increased miR-484 in prostate tumors associates with early disease recurrence, while miR-484 expression in human prostate cancer cells enhances cancer cell mobility. Using RNAseq and bioinformatics, we identified candidate target genes of miR-484 and generated a list of potential tumor suppressors. One candidate in this list was PSMG1. We applied luciferase assays and immunoblotting to confirm that miR-484 directly targets PSMG1. Additional *in vitro* assays with cancer cell lines showed that PSMG1 knockdown rescued the reduction in mobility brought on by miR-484 inhibition, pointing toward the existence of a miR-484–PSMG1 axis in prostate cancer.

CONCLUSIONS: We hypothesize that miR-484 is an oncogene in the prostate that increases cancer cell mobility, with PSMG1 being a mir-484 target in this process.

## Introduction

Since their initial discovery in *Caenorhabditis elegans* [1], microRNAs (miRs) have been reported to be active in numerous organisms, such as plants [2] and animals [3–5]. miRs reduce mRNA stability and translation, primarily by binding the 3′ untranslated region (UTR) of target transcripts [6], which leads to gene expression changes downstream of the miRs and may also alter cell biology. Thus, miRs and their targets play important roles in organismal development and functions.

An area of interest among researchers is the implication of miRs in diseases. For instance, miR-33 deficiency in mice has been noted to promote hyperlipidemia and obesity, but its deficiency in macrophages decreased lipid accumulation, reducing atherosclerotic plaques [7]. In another example of clinical relevance, miR-122 is a liver-specific miR, which modulates hepatitis C virus (HCV) abundance and replication [8–10]. Administration of miR-122 inhibitor in patients with HCV-reduced HCV RNA levels in a dose-dependent fashion [11], further demonstrating the clinical relevance of miRs.

Our laboratory has studied prostate carcinogenesis and identified miRs that are involved in disease progression. For instance, miR-1 is a candidate tumor suppressor [12], as its reduced expression predicts disease recurrence, and it is epigenetically silenced and among the most downregulated miRs in human prostate tumors. miR-1 probably opposes carcinogenesis by inhibiting proliferation and metastasis-associated invasion of cancerous cells and may act as a tumor suppressor through exosomal transfer. On the other hand, the miR-106b-25 cluster is a candidate oncogene [13] because its increased expression predicts early disease recurrence, while it also tends to be upregulated in human prostate tumors. miR-106b-25 was shown to promote carcinogenesis by decreasing apoptosis of cancerous cells, while increasing their focal adhesion to the basement membrane. In the present study, we used a publicly available database with miR expression data in prostate cancer [14], and applying stringent criteria, we identified another candidate oncogene, miR-484. We noted that miR-484 expression is directly associated with prostate cancer recurrence and found, using cell culture models, that miR-484 is a candidate oncogene by modulating mobility of prostate cancer cells.

## Materials and methods

### Cell lines

The following six human prostate cell lines were obtained from the ATCC (Manassas, VA): RWPE-1, RWPE-2, 22Rv1, LNCaP, DU145, and PC-3. These cell lines have been regularly authenticated using a short tandem repeat analysis with GenePrint10 and tested for Mycoplasma contamination.

### RNA extraction, miR-qPCR and qPCR

All reagents and equipment were from Thermo Fisher Scientific (Waltham, MA). Total RNA was isolated using TRIzol (Cat# 15596026) according to instructions.

Ten nanograms of total RNA were reverse transcribed using TaqMan MicroRNA Reverse Transcription Kit (Cat# 4366596). miR-qPCR assessing expression levels of miR-484 was performed in triplicate using TaqMan probes for miR-484 (Cat# 4427975, Assay ID 001821) and U6 snRNA (Cat# 4427975, Assay ID 001973) with TaqMan Universal Master Mix II, no UNG (Cat# 4440041). The U6 snRNA was used as the internal standard reference.

A thousand nanograms of total RNA were reverse transcribed using High-Capacity cDNA Reverse Transcription Kit with RNase Inhibitor (Cat# 4374967). qPCR was performed in triplicate using TaqMan probes for PSMG1 (Cat# 4331182, Assay ID Hs00186605_m1), TSPAN15 (Cat# 4351372, Assay ID Hs01057011_m1), SNRNP200 (Cat# 4331182, Assay ID Hs00391890_m1), USP18 (Cat# 4331182, Assay ID Hs00276441_m1) and 18s rRNA (Cat# 4331182, Assay ID Hs99999901_s1) with TaqMan Gene Expression Master Mix (Cat# 4369016). The 18s rRNA was used as the internal standard reference. Owing to the high abundance of 18s rRNA, total RNA was diluted 1:100 for the 18 s rRNA assay yielding average *C*_T_ 13-20.

Assays were run on MicroAmp Optical 384-Well Reaction Plate with Barcode (Cat# 4343814) and analyzed using 7900HT Fast Real-Time PCR System with 384-well block module (Cat# 4329001). Normalized expression was calculated using the comparative *C*_T_ method, and fold changes were calculated from 2^ΔΔ*C*T^ values for miR-484 or each gene [15].

### Cell proliferation

Human prostate cells were seeded at 2 × 10^5^ cells/well in six-well plates. Twenty-four hours after seeding, cells were transfected with 30 nM of *mir*Vana miRNA inhibitor, negative control #1 (Cat# 4464076), or *mir*Vana miR-484 inhibitor (Cat# 4464084, Assay ID MH10379) using Lipofectamine RNAiMAX (Cat# 13778150) from Thermo. Forty-eight hours after transfection, four columns of wells in a 96-well plate were seeded with each of the following: 100 μl of media/well with no cells, 1 × 10^3^ negative control-transfected cells/well or 1 × 10^3^ miR-484 inhibitor-transfected cells/well. Each of the four columns of wells represented eight replicates for a time point—Day 0, 1, 3 or 5. Normalized expression of miR-484 was calculated using the comparative *C*_T_ method, and fold changes were calculated from 2^ΔΔ*C*T^ values for miR-484. A 70–80% reduction of miR-484 induced by the inhibitor relative to the negative control was verified by miR-qPCR.

Cell viability was measured on Days 0, 1, 3 and 5 using alamarBlue Cell Viability Reagent (Cat# DAL1025) from Thermo with a FLUOstar Omega microplate reader from BMG Labtech (Ortenberg, Germany), using fluorescence (excitation at 544 and emission at 590 nm). Background fluorescence values of media-only wells were subtracted from the values of wells with negative control or miR-484 inhibitor-transfected cells. The subtracted fluorescence values of the eight replicates from negative control or miR-484 inhibitor-transfected cells on Days 0, 1, 3 and 5 were compared using unpaired *t* test with Welch’s correction in GraphPad Prism 7. The experiment was repeated for *n* = 3.

### Apoptosis

Cells were seeded at 1.6 × 10^6^ cells/well in T25 flasks with *n* = 3. Twenty-four hours after seeding, cells were transfected with 30 nM of *mir*Vana miRNA inhibitor, negative control #1 (Cat# 4464076) or *mir*Vana miR-484 inhibitor (Cat# 4464084, Assay ID MH10379) using Lipofectamine RNAiMAX (Cat# 13778150) from Thermo. Forty-eight hours after transfection, 1.2 × 10^6^ cells were harvested, and the cell pellets frozen at −80°C. Normalized expression of miR-484 was calculated using the comparative *C*_T_ method, and fold changes were calculated from 2^ΔΔC^*^T^* values for miR-484. A 70–80% reduction of miR-484 induced by the inhibitor relative to the negative control was verified by miR-qPCR.

The caspase-3 activity, a readout of apoptosis, was determined in cell pellets with EnzChek Caspase-3 Assay Kit #2 (Cat# E13184) from Thermo and measured using a FLUOstar Omega microplate reader from BMG Labtech, using fluorescence (excitation at 485 and emission at 520 nm). The caspase-3 values of negative control or miR-484 inhibitor-transfected cells were compared using unpaired *t* test with Welch’s correction in GraphPad Prism 7.

### Migration and invasion assays

Cells were seeded at 2 × 10^5^ cells/well in six-well plates. Two different transfection conditions were performed as follows:

1. Twenty-four hours after seeding, cells were transfected with 30 nM of *mir*Vana miRNA inhibitor, negative control #1 (Cat# 4464076), or *mir*Vana miR-484 inhibitor (Cat# 4464084, Assay ID MH10379) using Lipofectamine RNAiMAX (Cat# 13778150) from Thermo to assess the effect of miR-484 inhibition. Forty-eight hours after transfection, normalized expression of miR-484 was calculated using the comparative *C*_T_ method, and fold changes were calculated from 2^ΔΔ*C*T^ values for miR-484. A 70–80% reduction of miR-484 induced by the inhibitor relative to the negative control was verified by miR-qPCR.
2. Twenty-four hours after seeding, cells were transfected with the following: (1) 30 nM each of *mir*Vana miRNA inhibitor, negative control #1 (Cat# 4464076) and *Silencer* Select negative control #1 siRNA (Cat# 4390843); (2) 30 nM each of *mir*Vana miR-484 inhibitor (Cat# 4464084, Assay ID MH10379) and *Silencer* Select negative control #1 siRNA (Cat# 4390843); or (3) 30 nM each of *mir*Vana miR-484 inhibitor (Cat# 4464084, Assay ID MH10379) and PSMG1 *Silencer* Select siRNAs (Cat #4392420, IDs S16406 and S16407) using Lipofectamine RNAiMAX (Cat# 13778150) from Thermo to assess the effect of miR-484 and PSMG1 dual inhibition. Forty-eight hours after transfection, normalized expression of PSMG1 was calculated using the comparative *C*_T_ method, and fold changes were calculated from 2^ΔΔ*C*T^ values for PSMG1. Downregulation of miR-484 and upregulation of PSMG1 in response to *mir*Vana miRNA inhibitor transfection were verified by qPCR. Expression differences were compared with *Silencer* Select negative control siRNA.

For migration assay, 3 × 10^4^ cells for each treatment condition were seeded in replicate wells in the upper chamber of CIM plate 16 (Cat# 05665817001), whose bottom chamber contained media with 10% FBS as an attractant. The assembled chambers of CIM plate 16 were loaded onto xCELLigence RTCA Systems (Cat# 00380601050) from ACEA Biosciences (San Diego, CA), and cell movements from upper to bottom chambers were quantified in 15-min intervals for 48 h. The experiment was repeated for *n* = 3.

For invasion, the steps of migration assay were replicated with an additional step 48 h after transfection: the wells in the upper chamber of CIM plate 16 were coated with Matrigel Matrix (Cat# 356234) from BD Biosciences (Franklin Lakes, NJ), and the upper chamber was placed in a 37°C incubator for 4 h for the Matrigel to solidify. The experiment was repeated for *n* = 3. We used Matrigel at a 5% concentration.

### Immunoblotting

DU145 cells were transfected with 30 nM of *mir*Vana miRNA mimic, negative control #1 (Cat# 4464058), or miR-484 mimic (Cat# 4464066, Assay ID MC10379) from Thermo (Waltham, MA). Forty-eight hours after transfection, total protein was extracted from cells using RIPA buffer (Cat# 89901) with Halt Protease Inhibitor Cocktail (Cat# 87786), and 50 μg of protein was loaded on a 16% Tris-Glycine gel (XP00165BOX) from Thermo followed by immunoblotting. Primary antibodies used were PSMG1 (1:1000 of Cat# 13378) from Cell Signaling (Danvers, MA) and β-Actin (1:5000 of sc-47778) from Santa Cruz (Dallas, TX). The secondary antibody for the PSMG1 Western was an ECL anti-Rabbit IgG, HRP-linked F(ab')_2_, fragment (from donkey) (#NA9340V) from GE Healthcare (Marlborough, MA), used at a 1:5000 dilution. Visualization of β-actin using the labeled primary antibody from Santa Cruz does not require a secondary antibody. Chemiluminescent signal was captured on the Bio-Rad ChemiDoc Imaging System. The experiment was repeated for *n* = 3.

### RNA sequencing and the strategy to obtain gene targets of miR-484

DU145 and PC-3 cells were transfected with 100 nM of *mir*Vana miRNA mimic, negative control #1 (Cat# 4464058), or miR-484 mimic (Cat# 4464066, Assay ID MC10379) from Thermo in quadruplicates and submitted to RNAseq and analyzed to identify downregulated genes, the likely targets of the miR.

RNAseq was performed at the Sequencing Facility, Leidos Biomedical Research, Inc., Frederick National Laboratory for Cancer Research (Frederick, MD). Briefly, 500 ng of total RNA was used for library preparation with the TruSeq V3 chemistry kit from Illumina (San Diego, CA). Sequencing was performed on an Illumina HiSeq 2000 system. For each sample, we generated approximately 50 million paired-end read at 101 bp length. Reads were trimmed for both adapters and low-quality bases using the Trimmomatic software and then aligned with the reference human hg19 genome and gene annotation from the *Ensembl* database (*v70*) using the Tophat software. RNA mapping statistics were calculated using the Picard software, and the average uniquely aligned reads were approximately 90% for each sample. We subsequently used the RNA-Seq workflow module in Partek Genomics Suite 6.6 from Partek Inc. (St Louis, MO; http://www.partek.com/) and the R/Bioconductor package *DESeq2* to identify differentially expressed genes at FDR < 5% and a fold change (≥1.5) cut-off. Partek performed an analysis based on RPKM units (reads per kilobase per million mapped reads), and the gene counts were fitted to a negative binomial generalized linear model with *DESeq2*.

The TargetScan database (http://www.targetscan.org) was utilized to generate a list of predicted gene targets of miR-484.

To generate an overlap between the downregulated transcripts in miR-484 mimic-transfected DU145 or PC-3 cells (cutoff: *P* < 0.01 and ≤ −1.5 fold expression change [14]) and TargetScan-predicted targets for miR-484, the *Oliveros, J.C. (2007-2015)* Venny software (http://bioinfogp.cnb.csic.es/tools/venny/index.html) was applied, which is an interactive tool for comparing gene lists with Venn diagrams.

In order to narrow down the list of genes from the overlap, expression of genes in prostate tumor versus non-cancerous tissues from eight cohorts (Grasso [16], Lapointe [17], Singh [18], Taylor [14], TCGA [19], Tomlins [20], Wallace [21] and Yu [22]) was extracted from Oncomine (http://www.oncomine.org, December 2016, Thermo Fisher Scientific, Ann Arbor, MI). The 49 and 42 candidate genes from the DU145 and PC-3 gene lists, respectively, that overlapped with predicted TargetScan targets were then further compared with the list of genes from these eight cohorts; the ones showing overlap with genes that had greater expression in normal tissue versus tumor in ≥ 2 cohorts were selected and searched for using PubMed. By doing so, we aimed to further narrow down the list of potential tumor suppressors targeted by miR-484.

### Luciferase reporter assay

The TargetScan database (http://www.targetscan.org) was utilized to locate the putative binding site of miR-484 in PSMG1 3′ UTR. The information was used to generate pLenti-UTR-Luc PSMG1 WT and pLenti-UTR-Luc PSMG1 MUT for the luciferase assays.

The reporter construct, pLenti-UTR-Luc, consists of a CMV promoter followed by luciferase from *Photinus pyralis*, and it was digested with EcoRI and XhoI. The 3′ UTR sequences of PSMG1 WT and PSMG1 MUT were amplified by PCR from human gDNA plus cDNA as follows: WT was amplified by PCR with primers at both ends. The MUT was amplified using outer primers together with internal primers that carried the mutation. The overlapping sequences of the two internal primers allowed the two PCR amplicons to be assembled into 1 mutant 3′ UTR using a Ligation-Free Cloning kit (Cat# E001) from abm (Richmond, Canada). The pieces were joined using the Ligation-Free Cloning kit, inserting each 3′ UTR into the reporter construct. The sequences of constructs were verified prior to experiments.

### PSMG1 WT

TCTTAAACATTGTTTTGTAGTGTATATTACTTGTCCATTCCTTTAAGGGGAGCAGCCTGCACTCTTTTGTAGATTACTTTTGGGGGATATATTTTGAGAATGATGAAACGGAATAAAATTGTAAAAAATTAATTGTAGTTTTAA.

### PSMG1 MUT, MUTATED BASES

TCTTAAACATTGTTTTGTAGTGTATATTACTTGTCCATTCCTTTAAGGGGAGCTTTAAACACTCTTTTGTAGATTACTTTTGGGGGATATATTTTGAGAATGATGAAACGGAATAAAATTGTAAAAAATTAATTGTAGTTTTAA.

Seventy-thousand cells were seeded in 24-well plates and co-transfected with 100 nM of *mir*Vana miRNA mimic, negative control #1 (Cat# 4464058), or miR-484 mimic (Cat# 4464066, Assay ID MC10379) from Thermo, 100 ng of luciferase reporter pLenti-UTR-Luc PSMG1 WT or pLenti-UTR-Luc PSMG1 MUT, and 4 ng of pRL-CMV *Renilla* luciferase reporter (Cat# E2261) from Promega (Madison, WI), using TransIT-X2 (Cat# 6004) from Mirus (Madison, WI). Cells were cultured for another 48 h and washed once in PBS. Using a Luc-Pair Duo-Luciferase HS Assay Kit (Cat# LF004) from GeneCopoeia (Rockville, MD), cells were then lysed, loaded onto white 96-wells in quadruplicates, and their luciferase/*Renilla* ratios were measured using a FLUOstar Omega microplate reader from BMG Labtech. The experiment was repeated for *n* = 4.

### Statistical analysis and usage of public databases

Unpaired *t* test with Welch’s correction from GraphPad Prism 7.0 was used to assess the endpoints, including differences in miRNA and mRNA expression, apoptosis and luciferase assays. All statistical tests were two-sided, and *P* < 0.05 was considered significant. Kaplan–Meier analysis in GraphPad Prism 7.0 was utilized for comparing differences in disease-free survival between groups of patients divided by miR-484 or PSMG1 expression.

TCGA data on prostate cancer were downloaded from the cBio Cancer Genomics Portal (http://cbio.mskcc.org/cancergenomics/prostate/data/) to obtain expression of miR-484 between tumor and normal tissues of patients. Data from Taylor et al. *Cancer Cell* 2010 were downloaded from the NCBI and utilized to obtain miR-484 expression, PSMG1 expression and disease-free survival of patients according to the expression of miR-484 and PSMG1.

## Results

### miR-484 is inversely associated with disease-free survival and upregulated in human prostate tumors

**Figure 1:**
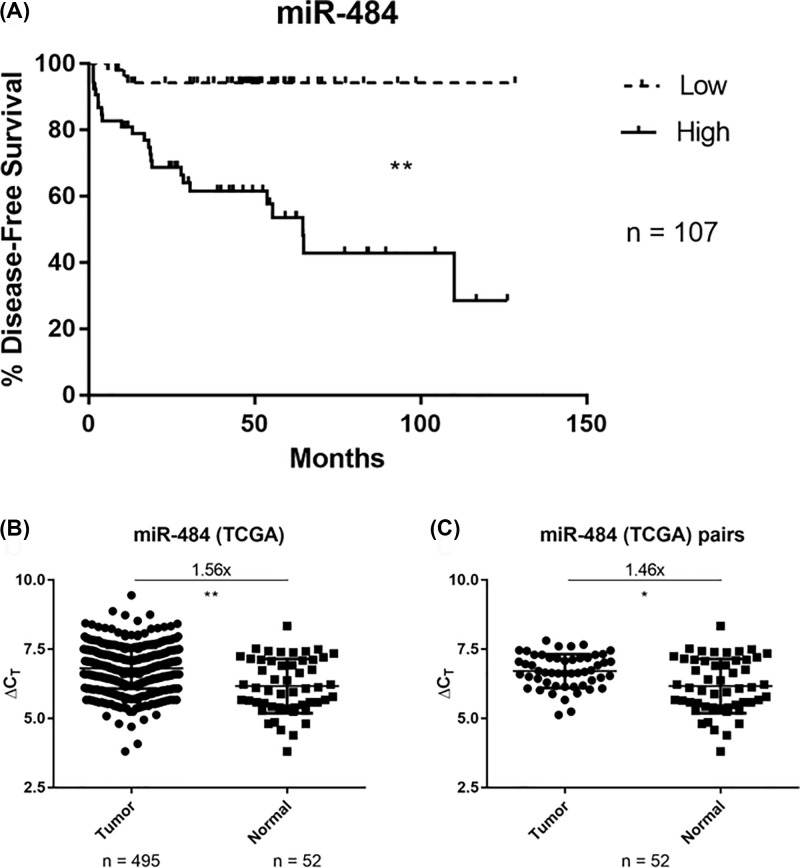
Association of high miR-484 with reduced disease-free survival and increased expression of miR-484 in human prostate tumors (**A**) Kaplan–Meier survival analysis showing that miR-484 expression is inversely associated with disease-free survival. Data were extracted from Taylor et al. *Cancer Cell* 2010. **P <0.0001 per log-rank test. (**B** and** C**) miR-484 shows higher expression in tumor versus normal in total (B) and in matching pairs (C). Normalized miR-484 expression data were extracted from TCGA, and fold differences were calculated using 2^ΔΔCT^. *P <0.05, **P <0.0001, unpaired *t* test with Welch’s correction.

We hypothesized that the miRs of interest in prostate carcinogenesis are the ones significantly related to survival of patients. Therefore, we assessed that miRs have the most robust relationships with survival in TCGA and Taylor datasets and identified miR-484 as being significantly associated with decreased disease-free survival (Figure 1A). This was intriguing because miR-484 has already been reported to be relevant in cancer biology, acting as a tumor suppressor in the colon [23,24] and as an oncogene in the liver [25]. Given the association of high miR-484 with decreased survival, we hypothesized that miR-484 is a candidate oncogene in the prostate. We then sought to compare the expression of miR-484 in prostate tumor versus normal tissues. Our expectation was that a candidate oncogene would be more highly expressed in the tumor. Indeed, miR-484 expression in tumors was higher than the expression in the non-cancerous prostate (Figure 1B,C). Therefore, the data from both survival and tissue expression analyses indicated that miR-484 is a candidate oncogene in the prostate.

### Gene targets of miR-484

**Figure 2:**
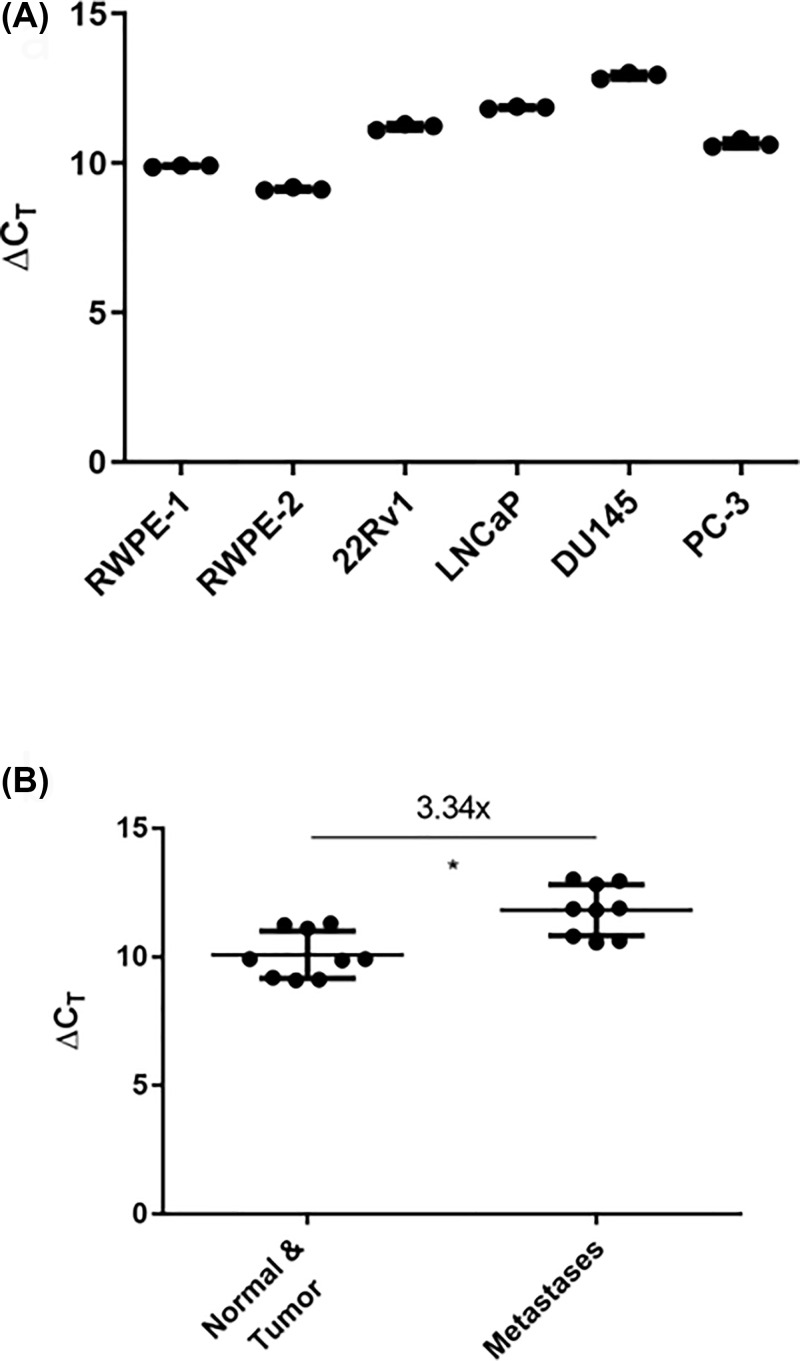
miR-484 expression is elevated in prostate cancer cells derived from metastatic origin compared with those derived from normal cells (RWPE-1) and primary tumor (RWPE-2, 22Rv1) (**A**) miR-484 expression in the six human prostate cell lines as assessed by miR-qPCR. *N* = 3 for each cell line with solid lines indicating the average miR expression. (**B**) miR-484 expression grouped by origin into three cell lines from normal and primary tumor (RWPE-1; RWPE-2 and 22Rv1) versus three cell lines from metastases (LNCaP, DU145 and PC-3). miR-484 expression fold differences between the two groups derived from the average miR expression in the two groups and calculated using 2^ΔΔ*C*T^. The error bars (± S.D.) are denoted within each group, and *P <0.05 using unpaired *t* test with Welch’s correction.

We assessed miR-484 expression by miR-qPCR in six human prostate cell lines (Figure 2A) and noted a greater expression in the cell lines derived from metastases compared with those from the non-cancerous prostate or the primary tumor (Figure 2B). Combining our hypothesis of miR-484 being a candidate prostate oncogene and its relatively higher expression in the cell lines from metastases, we reasoned that miR-484 may be more relevant in these cell lines than those from the primary tumor. Therefore, we decided to use the cell lines from metastases for our experiments.

In order to identify protein-coding gene targets of miR-484 in prostate cancer cells beyond the prediction provided by TargetScan, we transfected two human prostate cancer cell lines, DU145 and PC-3, with a miR-484 mimic and searched for the genes commonly downregulated by this approach using RNAseq analysis of miR-484 mimic and negative control miR-transfected cells. We chose DU145 and PC-3 cells because they are both derived from metastases and express the highest and the lowest level of miR-484 among the three metastatic cell lines (Figure 2A). By overexpressing miR-484 in two cell lines, we aimed to identify potential gene targets of miR-484 using our RNAseq approach.

**Figure 3:**
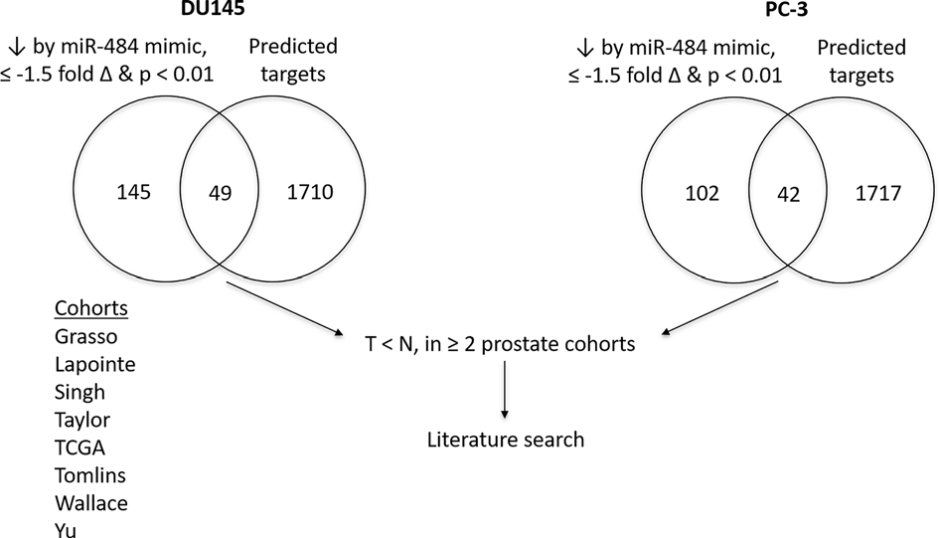
Scheme for finding tumor suppressors targeted by miR-484 RNAseq, TargetScan, Oncomine cohorts and literature search were utilized to identify potential tumor suppressors targeted by miR-484 in DU145 and PC-3 cells. The 49 and 42 candidate genes from the DU145 and PC-3 gene lists, respectively, that overlapped with predicted TargetScan targets were compared with the list of genes from eight prostate cancer cohorts. The ones showing overlap with genes that had greater expression in normal tissue versus tumor in ≥2 cohorts were selected and searched for using PubMed.

After analyzing the RNAseq data from the miR mimic-transfected cells, we then overlapped the list of downregulated genes with the list of predicted gene targets of miR-484 in TargetScan (Figure 3). We subsequently compared the 49 (for DU145) and 42 genes (for PC-3) from the overlap with TargetScan predictions (see Supplementary Table S1) against a list of candidate tumor suppressors (decreased expression in tumors when compared with non-cancerous tissue in two Oncomine datasets) and performed a literature search. By doing so, we aimed to combine the powers of RNAseq, TargetScan, Oncomine and literature searches to select tumor suppressors targeted by miR-484, as shown in Figure 3.

### miR-484 directly targets PSMG1

**Figure 4:**
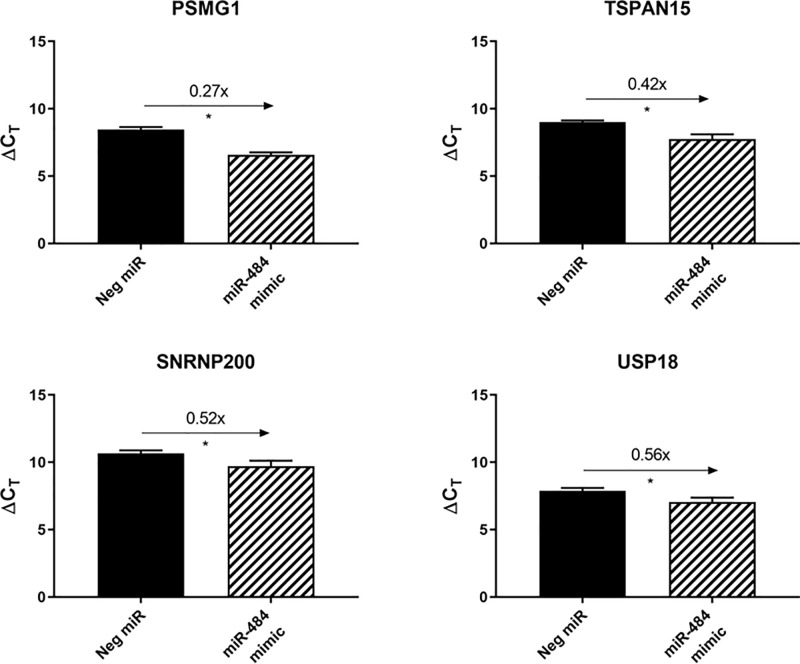
Downregulation of candidate miR-484 targets after transfection of DU145 cells with miR-484 miR-484 mimic was transfected into DU145 cells, which was followed by qPCR to measure expression of the predicted targets. Shown targets were selected from the gene list obtained from RNAseq data after transfection of DU145 cells with the miR-484 mimic. Top-ranked candidates were selected; *N* = 3. Fold differences compared with negative control miR calculated using 2^ΔΔCT^. *P <0.05 using unpaired *t* test with Welch’s correction.

The search for gene targets of miR-484 rendered several candidates, one of which was PSMG1; it showed the most significant change and had the greatest fold decrease (Table 1). This gene has been reported to be involved in proliferation and was found to be upregulated in several human cancer cell lines, including those from the breast, cervix and liver [26]. However, its relevance in prostate cancer has not been reported, which we wanted to clarify. Along with several other selected candidate targets of miR-484 from the search, PSMG1 showed the expected reduction in expression from miR-484 mimic transfection (Figure 4).

**Table 1:** Selected candidate gene targets of miR-484 in DU145 and PC-3

DU145			PC-3		
Log_2_Fold Δ	*P*-value	Gene	Log_2_Fold Δ	*P*-value	Gene
−1.62395	5.12E-294	**PSMG1**	−1.39889	1.08E-64	**PSMG1**
−1.33161	2.51E-08	SAMD11	−1.12007	6.80E-49	CRTC2
−1.27277	1.26E-164	CRTC2	−1.08363	1.08E-29	**TSPAN15**
−1.26587	3.95E-06	PAPPA2	−0.91939	1.51E-30	UPF3A
−1.10678	0.000728	RGS16	−0.91304	6.24E-16	**TMEM200B**
−1.06842	3.68E-105	**TSPAN15**	−0.73298	1.56E-17	**PBXIP1**
−0.92574	7.72E-17	**TMEM200B**	−0.72722	1.25E-08	**USP18**
−0.88175	8.84E-16	LGSN	−0.71117	1.20E-06	FAM46B
−0.88117	3.58E-228	**SNRNP200**	−0.69965	4.26E-05	NAT14
−0.83021	1.33E-38	**USP18**	−0.68631	2.22E-14	**PIM3**
−0.82767	6.10E-95	**TTPAL**	−0.68625	5.30E-14	**TTPAL**
−0.81904	0.000548	GRAMD2	−0.68543	1.65E-11	DAPP1
−0.78365	1.47E-26	HOMER1	−0.68167	2.86E-40	PPARD
−0.76809	4.52E-78	IGFBP4	−0.68	8.09E-08	**TSEN34**
−0.74344	3.57E-09	SNN	−0.65831	0.00142	DLX2
−0.69411	9.31E-56	**PIM3**	−0.64509	3.49E-21	**SNRNP200**
−0.67298	6.32E-28	ARL10	−0.62076	0.00049	BZRAP1
−0.66818	2.50E-05	KIAA1377	−0.60933	5.52E-05	SPATA2L
−0.64456	1.77E-17	**PBXIP1**			
−0.62842	0.005225	CNRIP1			
−0.61983	3.32E-25	TRIM56			
−0.61181	5.56E-47	GCNT2			
−0.61129	6.47E-43	**TSEN34**			
−0.5996	1.24E-23	ETV6			
−0.59027	3.61E-27	SEMA4D			
−0.59026	1.43E-11	JHDM1D			

The listed genes showed T < N in Oncomine prostate cohorts.

The candidates are organized by ascending fold change, from miR-484 mimic transfection.

The ones, which appeared in RNAseq for both DU145 and PC-3, are listed in bold.

**Figure 5:**
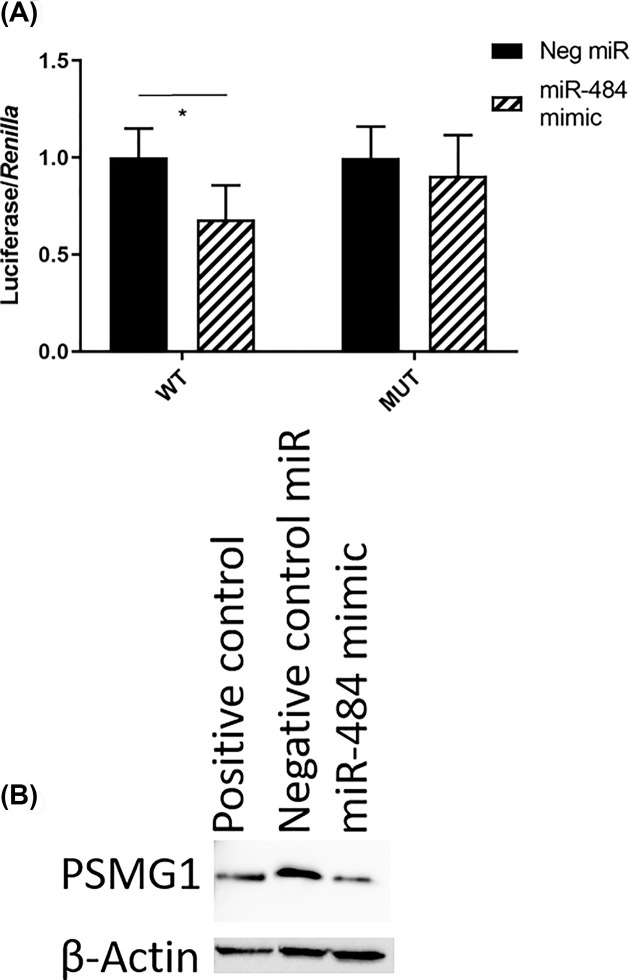
PSMG1 is directly targeted by miR-484 (**A**) Sequence-verified wild-type (WT) 3′ UTR or mutant (MUT) 3′ UTR of PSMG1 was cloned into a luciferase reporter plasmid and transfected into DU145 human prostate cancer cells with *Renilla* plasmid (for normalization) and with either negative control miR or miR-484 mimic. *Y*-axis indicates the ratio of luciferase to *Renilla*, relative to the miRNA mimic, when negative control was set to 1.0. *N* = 4 with error bars (± S.D.), and *P <0.05 using unpaired *t* test with Welch’s correction. (**B**) Transfection of DU145 cells with miR-484 mimic reduces PSMG1 protein expression. Shown are Western blot results with β-actin expression as a reference and loading control.

In order to confirm that PSMG1 is a direct target of miR-484, we transfected DU145 with miR-484 mimic and luciferase plasmids, which contained either the unaltered or mutated binding site of the miR in PSMG1 3′ UTR, and assayed luciferase activity (Figure 5A). We detected a significant reduction in luciferase activity upon transfection with luciferase plasmids containing wild-type (WT) compared with the mutated binding site of miR-484, which confirmed that PSMG1 is directly targeted by miR-484. We also observed that transfection of miR-484 mimic reduces PSMG1 protein level compared with non-targeting control (Figure 5B), so both luciferase assay and immunoblotting indicated that miR-484 directly targets PSMG1.

### miR-484 inhibition reduces cancer cell mobility, which is counteracted by PSMG1

**Figure 6:**
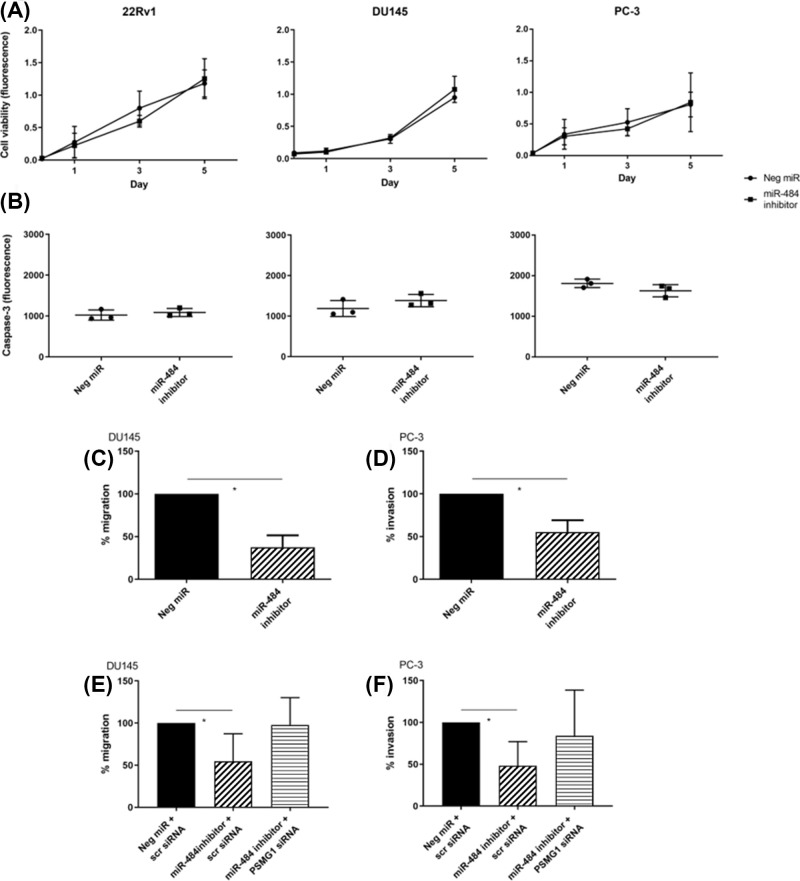
miR-484 inhibits mobility but not proliferation or apoptosis of human prostate cancer cell lines (**A**) miR-484 inhibition had no effect on the viability of 22Rv1, DU145 or PC-3 cells over 5 days. Cell viability was measured using fluorescence (*E*x/*E*m = 544/590 nm). Differences between miR-484 inhibitor and negative control miR were non-significant (NS) according to unpaired *t* test with Welch’s correction; *N* = 3 with error bars (± S.D.). (**B**) miR-484 inhibition had no effect on apoptosis of 22Rv1, DU145 or PC-3 cells. Apoptosis was measured 48 h after miR transfection using caspase-3 activity as a readout, which was measured using fluorescence (*E*x/*E*m = 485/520 nm); *N* = 3 with error bars (± S.D.). Differences between miR-484 inhibitor and negative control miR were NS according to unpaired *t* test with Welch’s correction. In contrast, miR-484 inhibition reduces migration (**C**) and invasion (**D**), which is rescued by inhibition of PSMG1 with siRNA (**E** and **F**). *Y*-axis denotes % migration or invasion relative to negative control at 48 h, set at 100%; *N* = 3 with error bars (± S.D.), and *P <0.05 using unpaired *t* test with Welch’s correction; Scr, scrambled.

We wanted to know whether miR-484 inhibition would reduce cancer cell proliferation. The reduction would confirm the hypothesized tumor suppressive role of PSMG1 [26] in reducing proliferation. However, we did not detect any reduction in cancer cell proliferation by miR-484 inhibition (Figure 6A), and neither for apoptosis possibly brought on by miR-484 inhibition (Figure 6B).

We lastly examined whether miR-484 may influence cancer cell migration and invasion, which are essential traits of metastasis. We found that miR-484 inhibition significantly reduced migration and invasion (Figure 6C,D). We further tested whether the co-transfection of PSMG1 siRNA and miR-484 inhibitor would rescue the phenotype. If found, this would show that miR-484 and PSMG1 are on the same axis that controls mobility. Indeed, we observed that the co-transfection rescues the phenotype, indicating that miR-484 and PSMG1 are on the same axis (Figure 6E,F).

## Discussion

miRs can act as oncogenes by directly targeting tumor suppressors, consequently downregulating them. An example of such includes miR-21, which acts as an oncogene in colon cancer by inhibiting Pdcd4 and consequently increasing invasion and metastasis [27]. Another example is miR-155, which acts as an oncogene in breast cancer by inhibiting SOCS1 and consequently increasing proliferation and inflammation [28]. Reports of miRs acting as oncogenes in prostate cancer have included miR-650, which suppresses CSR1 and consequently increasing metastasis [29], and miR-106a, which targets PTEN to promote proliferation, migration and invasion [30]. Thus, miRs can act as oncogenes in a variety of cancers, including prostate.

miR-484 has been reported to be active in a variety of conditions. For instance, it targets PCDH19 to decrease proliferation but increases neurogenesis in mice [31]. miR-484 acts as an oncogene in the liver by targeting SAMD9 to initiate tumorigenesis and cellular transformation through activated interferon pathway [25]. On the other hand, it works as a tumor suppressor in the colon by repressing CD137L, consequently reducing IL-8 production and cell viability of cancer cells [23]. In our experiments, miR-484 inhibition had no bearing on the proliferation of prostate cancer cells, which contrasts with miR-484 suppressing proliferation of cervical cancer cells [32], and increased proliferation of gemcitabine-resistant breast cancer cells [33]. Thus, its function and relation to cancer biology may be organ and context dependent.

In a meta-analysis of six miR datasets in prostate cancer, miR-484 was upregulated, which indicated it might be a negative prognostic biomarker [34]. In contrast, miR-484 was expressed lower in the serum of 72 patients with prostate cancer, compared with 34 controls without it [35]. Therefore, it is yet undetermined whether miR-484 is a positive or negative prognostic biomarker in prostate cancer.

In TCGA, we found that miR-484 is highly expressed in tumors compared with non-cancerous tissues, and it was inversely associated with survival in a publicly available database [14], which supported miR-484 as a negative prognostic biomarker in prostate cancer. We found that it directly targets PSMG1, and the reduced cell migration and invasion, brought on by miR-484 inhibition, are rescued by PSMG1 knockdown. Thus, we postulate that miR-484 acts as an oncogene in prostate cancer by targeting PSMG1 and affecting cell mobility. As PSMG1 was reported to be related to cell proliferation [26], its knockdown leading to increased mobility is a novel finding, potentially expanding its cellular functions.

We found several reports stating that PSMG1 increases susceptibility for inflammatory bowel diseases [36–38], which are linked to colon cancer [39–41]. However, we could not make a clear connection to cancer. We found reports of PSMG1 being related to cellular proliferation [26,42], but our results were not supportive of these findings. Otherwise, we did not find reports expounding its functions in cancer.

While the present work shows that miR-484 is a candidate negative prognostic biomarker in prostate cancer likely by targeting PSMG1 and modulating cell mobility, its conclusions are based on analyses from patient samples and *in vitro* experiments using cancer cell lines, which have limitations. Therefore, future murine *in vivo* data showing clear differences in metastasis brought on by miR-484 modulation would strengthen the conclusions. In addition, elucidation of functions of PSMG1, other than proliferation and mobility, relevant to carcinogenesis would complement the current findings and establish the importance of miR-484–PSMG1 axis in prostate cancer.

## Supplementary Material

Supplementary Table S1

## Abbreviations

HCV: hepatitis C virus
miR: microRNA
TCGA: The Cancer Genome Atlas
UTR: untranslated region
WT: wild-type
